# Composition and diversity analysis of the lung microbiome in patients with suspected ventilator-associated pneumonia

**DOI:** 10.1186/s13054-022-04068-z

**Published:** 2022-07-06

**Authors:** Dominic Fenn, Mahmoud I. Abdel-Aziz, Pouline M. P. van Oort, Paul Brinkman, Waqar M. Ahmed, Timothy Felton, Antonio Artigas, Pedro Póvoa, Ignacio Martin-Loeches, Marcus J. Schultz, Paul Dark, Stephen J. Fowler, Lieuwe D. J. Bos, The BreathDx Consortium, Waqar M. Ahmed, Antonio Artigas Raventos, Jonathan Bannard-Smith, Lieuwe D. J. Bos, Marta Camprubi, Luis Coelho, Paul Dark, Alan Davie, Emili Diaz, Gemma Goma, Timothy Felton, Stephen J. Fowler, Royston Goodacre, Craig Johnson, Hugo Knobel, Oluwasola Lawal, Jan-Hendrik Leopold, Ignacio Martin-Loeches, Tamara M. E. Nijsen, Pouline M. P. van Oort, Pedro Povoa, Nicholas J. W. Rattray, Guus Rijnders, Marcus J. Schultz, Ruud Steenwelle, Peter J. Sterk, Jordi Valles, Fred Verhoeckx, Anton Vink, Hans Weda, Iain R. White, Tineke Winters, Tetyana Zakharkina

**Affiliations:** 1 Department of Respiratory Medicine, Amsterdam UMC Location University of Amsterdam, Meibergdreef 9, Amsterdam, The Netherlands; 2 Department of Anaesthesiology, Amsterdam UMC Location University of Amsterdam, Meibergdreef 9, Amsterdam, The Netherlands; 3 Division of Immunology, Immunity to Infection and Respiratory Medicine, Faculty of Biology, Medicine and Health, University of Manchester, Manchester, UK; 4 Critical Care Department, CIBER Enfermedades Respiratorias, Corporación Sanitaria Universitaria Parc Taulí, Universitat Autonoma de Barcelona, Sabadell, Spain; 5 Hospital de São Francisco Xavier, Centro Hospitalar Lisboa Ocidental, CHRC, NOVA Medical School, New University of Lisbon, Estr. Forte do Alto Duque, 1449-005 Lisbon, Portugal; 6 Department of Intensive Care Medicine, Multidisciplinary Intensive Care Research Organization (MICRO), St. James’s Hospital, James’ St, Dublin, Ireland; 7 Department of Intensive Care, Amsterdam UMC Location University of Amsterdam, Meibergdreef 9, Amsterdam, The Netherlands

**Keywords:** Ventilator-associated pneumonia, Microbiome, Next-generation sequencing

## Abstract

**Background:**

Ventilator-associated pneumonia (VAP) is associated with high morbidity and health care costs, yet diagnosis remains a challenge. Analysis of airway microbiota by amplicon sequencing provides a possible solution, as pneumonia is characterised by a disruption of the microbiome. However, studies evaluating the diagnostic capabilities of microbiome analysis are limited, with a lack of alignment on possible biomarkers. Using bronchoalveolar lavage fluid (BALF) from ventilated adult patients suspected of VAP, we aimed to explore how key characteristics of the microbiome differ between patients with positive and negative BALF cultures and whether any differences could have a clinically relevant role.

**Methods:**

BALF from patients suspected of VAP was analysed using 16s rRNA sequencing in order to: (1) differentiate between patients with and without a positive culture; (2) determine if there was any association between microbiome diversity and local inflammatory response; and (3) correctly identify pathogens detected by conventional culture.

**Results:**

Thirty-seven of 90 ICU patients with suspected VAP had positive cultures. Patients with a positive culture had significant microbiome dysbiosis with reduced alpha diversity. However, gross compositional variance was not strongly associated with culture positivity (AUROCC range 0.66–0.71). Patients with a positive culture had a significantly higher relative abundance of pathogenic bacteria compared to those without [0.45 (IQR 0.10–0.84), 0.02 (IQR 0.004–0.09), respectively], and an increased interleukin (IL)-1β was associated with reduced species evenness (*r*_s_ = − 0.33, *p* < 0.01) and increased pathogenic bacteria presence (*r*_s_ = 0.28, *p* = 0.013). Untargeted 16s rRNA pathogen detection was limited by false positives, while the use of pathogen-specific relative abundance thresholds showed better diagnostic accuracy (AUROCC range 0.89–0.998).

**Conclusion:**

Patients with positive BALF culture had increased dysbiosis and genus dominance. An increased caspase-1-dependent IL-1b expression was associated with a reduced species evenness and increased pathogenic bacterial presence, providing a possible causal link between microbiome dysbiosis and lung injury development in VAP. However, measures of diversity were an unreliable predictor of culture positivity and 16s sequencing used agnostically could not usefully identify pathogens; this could be overcome if pathogen-specific relative abundance thresholds are used.

**Supplementary Information:**

The online version contains supplementary material available at 10.1186/s13054-022-04068-z.

## Introduction

Ventilator-associated pneumonia (VAP) develops in patients who have been mechanically ventilated for more than 48 h [1, 2] and is associated with high morbidity and health care costs [1, 3, 4]. Early diagnosis and treatment is critical [5], but the clinical diagnosis of VAP remains a challenge. Non-specific symptomology, clinical scoring systems and radiological features susceptible to inter-rater variability [6, 7] can lead to overdiagnosis and inappropriate use of antibiotics.

16s rRNA gene amplicon analysis is widely used for studying the lung microbiome by profiling bacterial composition and diversity in a sample [8, 9]. Named after the target gene, it relies on conserved and highly variable regions of the 16s rRNA gene found in all prokaryotes to identify bacteria [10, 11]. Using this technique, the relative abundance of gut bacteria in the lung of critically ill patients has consistently been shown to be increased in patients with acute respiratory distress syndrome (ARDS) [9]. Furthermore, changes in lung microbiome composition are linked to an altered host response and might explain the development of lung injury [12, 13].

From an ecological perspective, pneumonia has been described as an﻿ abrupt and emergent disruption in the complex homeostasis of such microbiota [14]. However, data from patients with VAP have been compared only to that from uninfected control patients on the intensive care unit (ICU) and not to patients in whom clinical suspicion of VAP had arisen. A better understanding of the composition of the lung microbiome in this clinically relevant population could further extend our understanding of VAP development and facilitate identification of patients who have bacterial pneumonia. When further developed, genomic techniques could help determine which patients should receive broad spectrum antibiotics while awaiting microbiological confirmation [1, 6]. However, studies evaluating its diagnostic capabilities are limited [15–17] with a lack of alignment on etiological diagnostic markers [16, 18].

Using bronchoalveolar lavage fluid (BALF) from ventilated adult patients suspected of VAP, we hypothesised that the lung microbiome differs between patients with positive and negative cultures. We aimed to link these changes in composition and diversity of the lung microbiome to alveolar inflammatory response. Last, we hypothesised that microbial composition is aligned with conventional cultures of commonly associated VAP bacteria.

## Methods

### Design, subjects and setting

This is a *post hoc* analysis of the ‘Molecular Analysis of Exhaled Breath as Diagnostic Test for Ventilator–Associated Pneumonia’—study (BreathDx), as described by van Oort et al. [19, 20]. BreathDx was an international multicentre, prospective observational cohort study of intubated and ventilated patients suspected of VAP. Patients were recruited between February 2016 and February 2018 from four ICUs: the Amsterdam University Medical Centers (UMC)—location Academic Medical Center (AMC), Amsterdam, the Netherlands; Manchester University NHS Foundation Trust—Wythenshawe Hospital (WH), Manchester University NHS Foundation Trust—Manchester Royal Infirmary (MRI) and Salford Royal NHS Foundation Trust (SRFT), Manchester, UK. Inclusion criteria were (1) 18 years and older and (2) intubation and mechanical ventilation for > 48 h and (3) clinical suspicion of VAP. Suspected VAP was defined by (1) systemic signs of infection [temperature > 38 or < 36.5 °C; white blood cell count < 4000 or > 12,000/mm^3^, purulent tracheal secretions], and (2) new infiltrates on chest X-ray [19]. Patients were excluded if they: (1) were deemed clinically inappropriate to collect samples from (e.g. end-of-life care); or (2) were in strict isolation (e.g. Middle East respiratory syndrome, Ebola or resistant tuberculosis). For this current analysis, samples were selected if data from both BALF semi-quantitative cultures and 16s rRNA analysis were available. Patient assent at the time of inclusion was obtained from a designated consultee with deferred written consent taken from patients who regained capacity, as previously outlined in the study protocol [20]. The study was approved by respective institutional review boards and registered by the UK Clinical Research Network (ID no. 19086).

### Study procedure and sample collection

Patients were included, and BALF samples were collected within 24 hours of the clinical suspicion of VAP using either a directed or non-directed broncho-lavage approach. Directed BAL was performed following BTS guidelines [21]. Non-directed BAL was performed by connecting a syringe to a 50-cm suction catheter before 20 ml 0.9% saline was injected into the patient’s airway. An aspirate of at least 4 ml was collected and aliquoted for: (1) routine culturing and (2) storage at − 80 °C for 16s rRNA sequencing once the study had finished. While BALF recovery was to be performed prior to the initiation of antibiotics, delaying treatment for the purpose of the study would have not been ethically permissible. Consequently, BALF may have been collected while patients were just started on antibiotics.

## Reference standard and pathogen selection

A positive (non-directed) BAL culture with a cut-off of ≥ 10^4^ CFU/ml was used as the primary reference test, as described previously [19]. Pathogen identification accuracy was only tested in the following pathogens: *Pseudomonas aeruginosa*, *Staphylococcus aureus*, *Klebsiella pneumoniae* and *Haemophilus influenzae*. Other causative pathogens were also found but in lower frequency limiting the assessment of diagnostic accuracy. However, the selected pathogens represented the majority of clinically relevant organisms associated with VAP [6, 22, 23] that are commonly encountered in patients suspected of VAP in North-Western Europe, where the study was conducted.

### Sample processing and 16s rRNA gene sequencing.

Duplicate PCRs of the 16s rRNA gene region V4–V5 were performed as described before [24, 25]. In brief, the PowerFecal DNA Kit (Qiagen, Venlo, Netherlands) was used for DNA extraction of BALF before PCR amplification using the Illumina MiSeq platform (CGEB-Integrated Microbiome Resource, Halifax, Canada) and primer pair 515F/926R was performed [26, 27]. The sequencing facility was kept unaware of patient clinical status or diagnosis. Further details on RNA extraction and amplicon library preparation are provided in the online supplement (see Additional file 1). The DADA2 pipeline and amplicon sequence variant (ASV) table generation was selected over the more conventional operational taxonomic unit (OTU) approach due to: (1) the increased downstream resolution, sensitivity and specificity [27, 28]; (2) data set independency of ASVs enables easier extrapolation to other studies and theoretical formulation of a clinical cut-off value [28]. The EzBioCloud database (version May 2018) was used to assess taxonomic classification of the identified ASVs.

Compositional variance is described by alpha and beta diversity indices, while taxonomic profiling provides details on microbial community and pathogen presence. The average relative abundance of pathogenic bacteria was estimated per patient sample by firstly selecting for genera that were only identified by both 16s rRNA analysis and conventional BALF culture. 16s sequencing-derived relative abundance for each genus was then summed to give a total relative abundance of pathogens per sample.

### Additional biomarker analysis

Interleukin (IL)-1b and IL-8 concentrations in BALF were estimated using enzyme-linked immunosorbent assay (ELISA). Commercial ELSIA kits from R&D Systems Inc. (Bio-Techne, Minneapolis, USA) were used according to manufacturer’s protocols.

### Study endpoints

﻿The primary endpoint of this study is the diagnostic accuracy of microbial composition and diversity analysis for patients suspected of VAP with and without a positive BALF culture. Secondary endpoints are: (I) the association between microbial diversity and composition and local inflammatory response, (II) the pathogen concordance between 16s sequencing and conventional culture, and (III) the diagnostic accuracy of 16s sequencing for the presence of the selected bacteria in culture.

### Sample size and calculation

The sample size calculation for the BreathDx study has previously been described and predetermined based on the development of a novel diagnostic test to exclude VAP and allow clinicians to withhold antibiotic treatment [19, 20]. For this *post hoc* analysis, no formal sample size calculation was performed—instead, the number of available patients served as the sample size.

### Statistical analysis

Statistical analysis was performed in R (version 3.6.1) through the R studio interface. Downstream analysis of 16s rRNA was performed using the *vegan* package within R to assess community composition and ASV level differences [29]. Diversity and composition data were compared between patient groups using the Mann–Whitney U test. To determine whether the differences in BALF microbiota were driven by culture positivity, beta diversity testing was performed using a permutational multivariate analysis of variance (PERMANOVA). The PERMANOVA was performed using Bray–Curtis dissimilarity matrix and BALF culture result as the dependent variable. The following co-variates as identified by Carney et al. were included: age, gender, study site and disease severity [Clinical Pulmonary Infection Score (CPIS) and (Acute Physiology and Chronic Health Evaluation II score (APACHEII)] [30] and days of mechanical ventilation (MV) before clinical suspicion of VAP. To assess diagnostic potential of diversity measures, the area under the receiver operating characteristics curve (AUROCC) was used. Spearman’s rank correlation was used to assess (I) the correlation between alpha diversity measures and inflammatory biomarkers (IL-1b and IL-8) and (II) the presence of pathogenic bacteria and inflammation. For the pathogen identification and specific analyses, AUROCC was calculated and used to identify an optimal relative abundance threshold with a predefined sensitivity of at least 99% [20]. Using this cut-off, the BALF relative abundance was then dichotomised before two-by-two contingency tables were constructed for each pathogen. Diagnostic test characteristics are reported for this cut-off [sensitivity, specificity, positive predictive value (PPV) and negative predictive value (NPV)]. Specificity and predictive values of 16s rRNA bacterial analysis were calculated with 95% binomial confidence intervals.

## Results

### Sample and patient characteristics

One hundred and eight patients suspected of VAP were recruited over the study period. Sufficient BALF for both semi-quantitative and 16s rRNA culture was available from 91 patients (83.3%). 16s rRNA sample depth was assessed, and one sample was removed due to inadequate bacterial count. Of the remaining 90 patients, 37 (41%) had positive BALF cultures. Patient characteristics are summarised in Table 1. The remaining patients were not included in the analysis (Additional file 1: Table S1 and Fig. S1). A similar time frame of mechanical ventilation and the clinical suspicion of VAP was observed in both patient groups. At the time of inclusion, BALF was to be collected prior to the initiation of antibiotics; however, for some patients antibiotics may have been started before BALF recovery due to clinical need. Data regarding this were unavailable for the analysis. After quality control and processing, 953,975 reads were obtained from the 90 samples, resulting in over 5000 individual ASVs with an average of 4743 reads per sample (IQR 1244–12,182 reads). Negative control samples (*N* = 2) of saline solution were included in the sequencing to identify potential contaminants. Bioinformatic processing showed minimal contamination with little to no reads detectable (Additional file 1: Analytical pipeline, Fig. S2 and Table S2). Prior to downstream analysis, ASVs were filtered using a threshold of 0.001% of the total reads before being assigned to taxonomic rank.

**Table 1:** Patient characteristics

	Culture negative (*N* = 53)	Culture positive (*N* = 37)	*p*
Age, median (IQR) yrs	59.0 (48–68)	56.5 (38.0–66.5)	0.275
Male, *n* (%)	35 (66.0)	23 (62.2)	0.877
Days on MV^*^, median (IQR)	8 (4.5–12.5)	7 (5–10)	0.697
Admission type, *n* (%)			0.218
Medical	29 (54.7)	13 (35.1)	
Planned surgical	10 (18.9)	11 (29.7)	
Emergency surgical	13 (24.5)	13 (35.1)	
Unrecorded	1 (1.9)	0	
Trauma, *n* (%)	12 (22.6)	18 (48.6)	0.019
Neurosurgery, *n* (%)	11 (20.8)	12 (32.4)	0.315
COPD, *n* (%)	8 (15.1)	5 (13.5)	1.000
ARDS, *n* (%)	3 (5.7)	0 (0.0)	0.381
APACHEII score, median (IQR)	21 (15–24)	14 (10–20)	0.009
CPIS score, median (IQR)	5 (4–6)	7 (6–7)	< 0.001
*T*_max_, °C, median (IQR)	38 (37–39)	38 (37–38)	0.551
WCC, median (IQR) 10^9^/ml	15.5 (11.3–21.0)	13 (11–17)	0.074
PaO_2_/FiO_2 max_, median (IQR) mmHg	240 (180–283.5)	232.5 (168.8–283.1)	0.85
P_max_, median (IQR) cmH_2_O	20 (16–25)	21 (16–24)	0.874
PEEP, median (IQR) cmH_2_O	8 (5–10)	8 (5–10)	0.983
Tidal volume, median (IQR) ml	479 (429.5–580)	538 (449–620)	0.298
Genus culture results**, *n* (%)			
Enterobacter		2 (5.4)	
Escherichia		2 (5.4)	
Haemophilus		4 (10.8)	
Klebsiella		4 (10.8)	
Pseudomonas		10 (27.0)	
Serratia		1 (2.7)	
Staphylococcus		13 (35.1)	
Stenotrophomonas		1 (2.7)	
ICU LOS, median (IQR) days	18 (14.0–26.8)	22 (16–35)	0.154
Hospital LOS, median (IQR) days	26.5 (15.5–42.8)	34 (21.8–64.5)	0.284
ICU mortality, *n* (%)	12 (22.6)	4 (10.8)	0.244

*ARDS* acute respiratory distress syndrome, *APACHE* acute physiology and chronic health evaluation, *CPIS* clinical pulmonary infection score, *Tmax* maximum temperature, *WCC* white cell count, *FiO2 max* maximum inspired fraction of oxygen ratio, *Pmax* maximum airway pressure, *PEEP* positive end-expiratory pressure, *LOS* length of stay

^*^Days on mechanical ventilation (MV) until VAP suspicion

^**^Potentially > 1 cultured pathogen per patient

Eight individual genera were isolated using cultures (Table 1), whereas 16s rRNA analysis was able to detect over 80 using a predefined sensitivity threshold of 95%. The top three identified genera by conventional culture were Staphylococcus (35%) *Pseudomonas* (27%) and *Haemophilus* (11%). Similarly, for patients with a positive BALF culture, the three most abundant genera found using 16s sequencing were identical (see Additional file 1: Fig. S3). In patients with a negative BALF culture, 16s rRNA analysis detected *Haemophilus*, *Enterococcus* and *Prevotella* as the most abundant genera (Additional file 1: Fig. S3).

### Diversity analysis

**Fig. 1:**
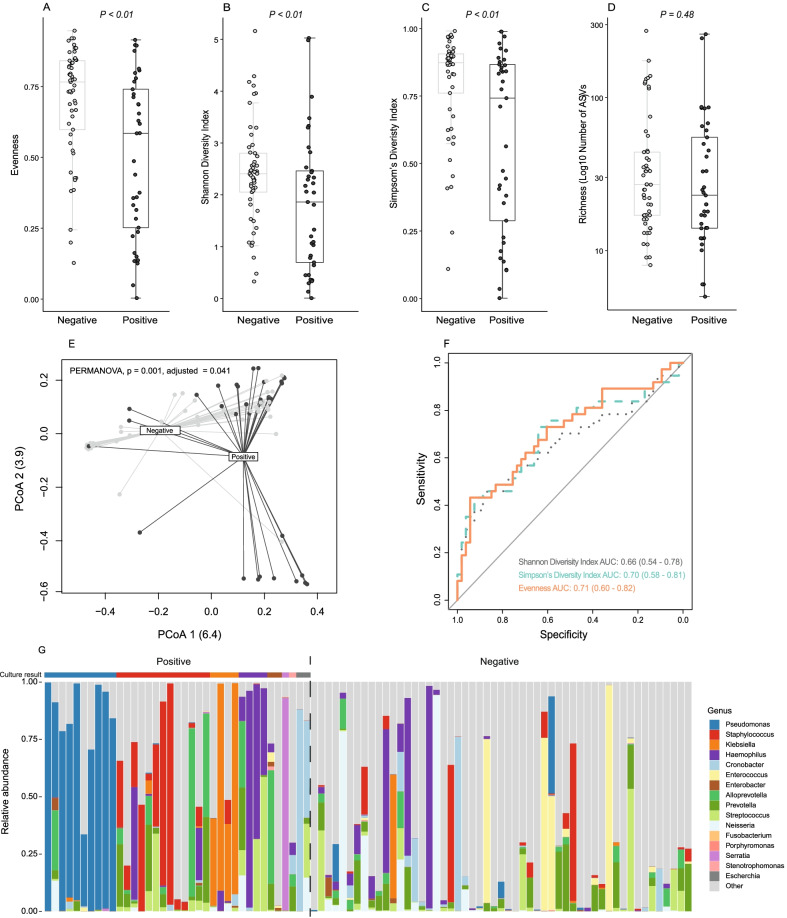
Lung microbiota is altered in patients with VAP (**A**–**C**) compared with (*N* = 37) patients without (*N* = 53) but showed no difference in compositional richness (**D**). Principal coordinate analysis showed significant differences between the microbial composition of patients with and without VAP (**E**), the X-axis indicating principal coordinate (PCoA) 1 and the Y-axis PCoA 2 on Bray–Curtis dissimilarity measure of 16S microbiome data. Despite significant dysbiosis in patients with VAP, compositional variance could not reliably diagnose VAP (**F**) when comparing evenness, Simpson’s diversity index and Shannon diversity index as predictor variables and in BALF culture as the outcome for all patients (*N* = 90). Individual patient bar plots split by VAP diagnosis compared with BALF culture-dependent result (**G**). The relative abundance of the top 10 genera along with all pathogens identified by semi-quantitative culture for each sample is shown. “Other” genus combines remaining genera in each sample

Comparing the alpha diversity measures at the ASV level between patients with and without positive BALF culture, there was a significantly decreased diversity for patients with a positive culture compared to those without (*p* < 0.01, Fig. 1, panels A–C). There was, however, no difference in richness (*p* = 0.48, Fig. 1, panel D). PERMANOVA testing showed a significant difference in beta diversity between patients with and without a positive culture (*p* = 0.001, adjusted *p* = 0.04, Fig. 1 panel E) after correction for possible confounders (age, gender and study site). Despite the observed diversity differences gross compositional variance using alpha diversity could not reliably discriminate culture positivity (AUROCC range 0.66–0.71, Fig. 1, panel F).

**Fig. 2:**
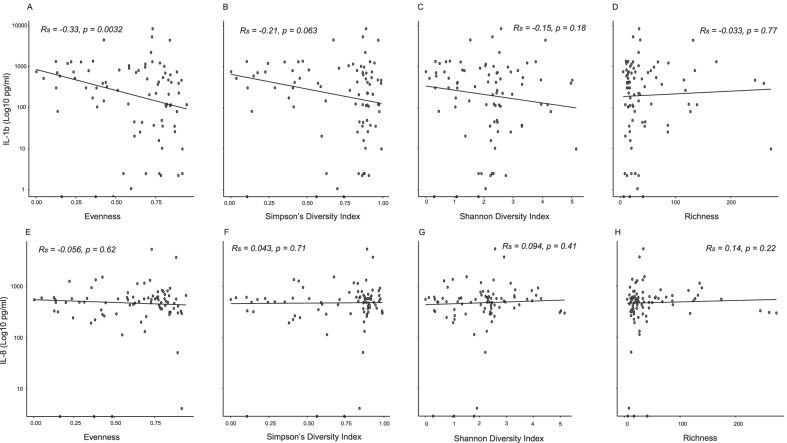
Spearman’s rank correlation analysis between alpha diversity measures (evenness, Simpson’s diversity index, Shannon diversity index and richness) and interleukin (IL)-1b (panel **A**–**D**) or IL-8 (panel **E**–**H**) expression

There was a significant negative correlation between IL-1b and evenness (*R* = − 0.33, *p* < 0.01, Fig. 2 panel A). However, no additional significant correlation was observed between the remaining diversity indices (Fig. 2, panel B–H).

### Microbiota compositional and relative abundance of pathogens

The majority of patients with positive culture showed increased genus dominance contributing to the decreased diversity observed when compared to patients with a negative culture (Fig. 1, panel G). For these patients, genus dominance was particularly evident for *Pseudomonas* and *Klebsiella.*

**Fig. 3:**
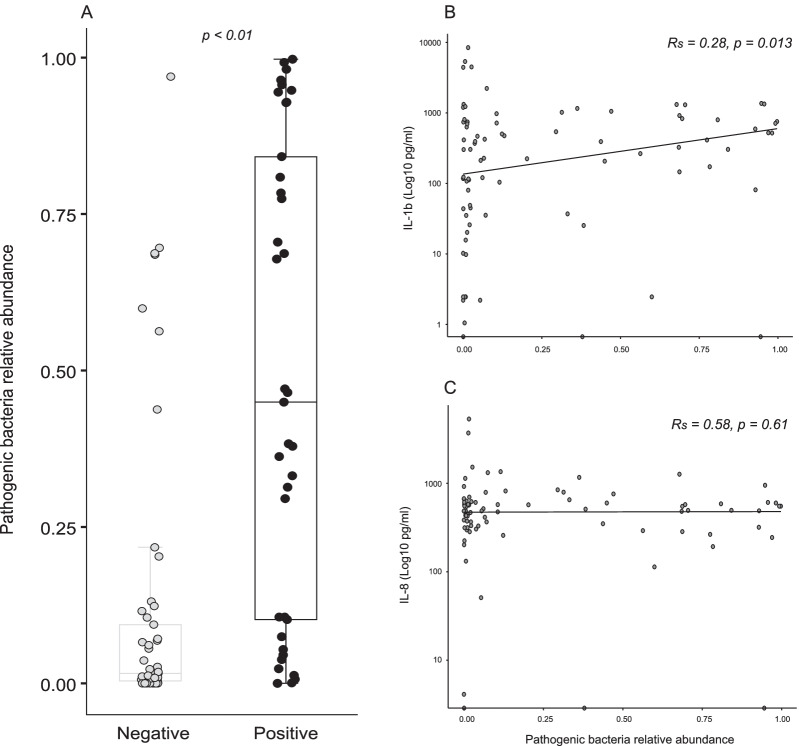
﻿Pathogenic bacteria relative abundance per patient sample (*N* = 90) is altered in patients suspected of VAP with positive BALF cultures compared to those with negative cultures (**A**). Spearman’s rank correlation analysis showed a positive correlation between pathogenic bacteria relative abundance and IL-1b expression (**B**) but not for IL-8 release (**C**)

Patients with positive BALF cultures had a significantly higher relative abundance of pathogenic bacteria compared to patients with negative cultures [culture positive: 0.45 (IQR 0.10–0.84), culture negative: 0.02 (IQR 0.004–0.09), *p* < 0.01, Fig. 3, panel A]. A positive correlation was found between the relative abundance of pathogenic bacteria and IL-1b (*r*_s_ = 0.28, *p* = 0.013, Fig. 3, panel B), but there was no significant correlation with IL-8 (*r*_s_ = 0.06, *p* = 0.61, Fig. 3, panel C).

### Concordance in identification of pathogens

**Fig. 4:**
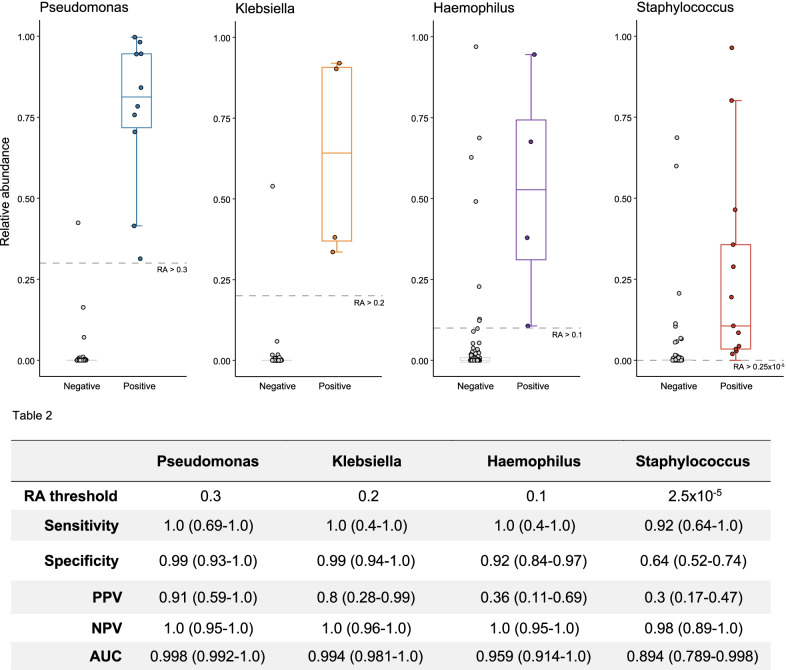
Relative abundance (RA) is depicted, comparing BALF culture positivity for selected pathogens with their identified diagnostic threshold. For each pathogen, sensitivity, specificity, positive predictive value (PPV), negative predictive value and area under the curve (AUC) are given with BALF conventional culture as the primary reference point and the identified 16s sequencing diagnostic relative abundance cut-off as the predictor. Values given with 95% binomial confidence intervals

Of the 37 patients with positive BALF cultures, 16s sequencing was able to correctly identify the causative pathogen in 33 (89.2%) samples while using a minimal relative abundance threshold of > 1% to define pathogen presence over potential background contamination. However, it failed to correctly identify the pathogen in 4 (10.8%) positive cultures and could not detect *Escherichia Coli* (Fig. 1 panel G). Conversely, of the patients with negative BALF cultures, using the same minimal relative abundance threshold (> 1%), one or more pathogenic bacteria were detected by 16s analysis in 35 (66%) of the 53 samples, albeit with a reduced relative abundance (Fig. 3, panel A). To further explore the diagnostic potential of 16s rRNA analysis, it was compared to the culture results for each of the selected pathogens. Pathogen concordance and identification performed better when considering each pathogen individually (AUROCC range 0.89–0.998, Fig. 4). Using the pre-specified high sensitivity needed for clinical application with high negative predictive value, the following relative abundance cut-offs were identified: *Pseudomonas* = 0.3; *Klebsiella* = 0.2, *Haemophilus* = 0.1 and *Staphylococcus* = 2.5 × 10^−5^. The sensitivity, specificity and predictive values of BALF 16s rRNA analysis are shown in Fig. 4—Table 2. Sensitivity and PPV were relatively good for *Pseudomonas* and *Klebsiella* but were much lower for *Staphylococcus* (PPV 30%; 95% CI 17–47%) and for *Haemophilus* (PPV 36%; 95% CI 11–69%).

## Discussion

In this study, patients with suspected VAP and a positive culture had increased dysbiosis of lower airway microbiome and an increased prevalence of pathogenic bacteria compared to patients who were suspected of VAP but had negative cultures. Furthermore, based on the studied culture-independent bacterial analysis, a decreased species evenness and an increased presence of pathogenic bacteria were associated with an increased local inflammatory response indicated by a rise in IL-1b concentration. However, measures of microbial diversity were insufficiently accurate to discriminate culture positivity, and agnostic pathogen detection using 16s rRNA sequencing was limited by high number of false positives. An alternative approach based upon pathogen-specific relative abundance diagnostic thresholds obtained from 16s sequencing showed an improved diagnostic accuracy.

We observed that the lung microbiome of patients suspected of VAP with a positive culture had more pathogenic genus dominance and decreased diversity. This is in line with the description of pneumonia from an ecological perspective, in which loss of eubiosis and diversity are ecological indications for pathogenic bacterial overgrowth and central events in the development of infection [14]. This finding extends the results of several previous studies into a population in which all patients were suspected of having VAP [18, 31–34]. It has been suggested that Shannon diversity index of the microbiome in tracheal aspirates was a good diagnostic marker for lower respiratory tract infections [35], but we could not replicate this diagnostic accuracy. Furthermore, the hereby presented evidence demonstrated that measures of microbial diversity were insufficiently accurate to predict culture positivity. Importantly, our study differs from all previous reports in that only patients with a clinical suspicion of VAP were included, resulting in a control group more representative of the real-life scenario where clinical decisions must be made.

Patients with more dysbiosis and loss of diversity also had higher levels of IL-1b in BAL fluid. This positive association could be related to the increased activation of the inflammasome complexes as demonstrated by ﻿Trachalaki et al. [36]. Critical to host immunity, inflammasomes mediate the caspase-1 inflammatory pathway responsible for the maturation and release of IL-1b, a potent proinflammatory cytokine [37–39]. This relation was confirmed in the current study and might elucidate a treatable association of microbiome dysbiosis, elevated caspase-1 activity and lung injury [9, 38]. Similar correlations were not observed for IL-8. The proximity of BALF sampling to the disease onset (within 24 hours) may provide an explanation for this, in that IL-1b is released to stimulate leucocyte activation immediately after pulmonary insult, whereas IL-8 is expressed later to aid leucocyte reprogramming as the disease state progresses [40, 41].

A good concordance was observed for identification of the genus of the causative pathogen between 16s rRNA analysis and conventional culture. This is in line with studies by Emonet et al. [16] and Miao et al. [42], which demonstrated that molecular analysis accurately identified bacteria grown by conventional culture. However, despite this, the diagnostic performance of untargeted 16s sequencing in the present study was limited by a high number of false positives, making clinical application of an unbiased approach unlikely. To overcome this, previous studies have suggested that pathogen dominance, as represented by an individual species’ relative abundance tenfold greater than any other microbes, could be used [42]. However, for this study some patients 16s rRNA detection of individual genus, even if non-dominant, was associated with a positive BALF culture. Consequently, this method could lead to an increased number of false negatives in our cohort.

In light of this, an alternative approach was evaluated using pathogen-specific relative abundance thresholds. We found that 16s sequencing was better suited to exclude *Pseudomonas* and *Klebsiella* presence. However, for *Staphylococcus* and *Haemophilus* the cut-offs were less reliable. This is in line with results from a similar comparison in invasively ventilated COVID-19 patients [43]. This could be a result of reduced taxonomic resolution of 16s sequencing: limited to genus rather than species level. Consequently, all species with a shared genus, including those other than *Staphylococcus aureus* and *Haemophilus influenzae*, were incorporated, potentially over representing pathogen presence. This is likely to limit the role 16s sequencing might have to pathogen exclusion in VAP. In addition, selecting a satisfactory diagnostic cut-off for *Staphylococcus* proved to be the most challenging. Unlike other pathogens, patients with a very low, even undetectable relative abundance had positive a BALF culture. This is likely due to the rigid cell wall of *Staphylococcus aureus* that makes DNA extraction difficult [44]. Alternative sequencing methods have been developed to maximise its detection [44]; however, such optimisation was not performed in the current study.

A strength of this study was the international multi-centred design and inclusion of patients only with clinical suspicion of VAP. We performed a comprehensive culture-independent analysis and conventional culturing for selected pathogens. Although the BreathDx study protocol and methodology were predefined and published [20], this was a post hoc analysis with no formal sample size calculation. The comparisons between culture positive and negative patients were sufficiently powered, but the pathogen-specific analyses were hampered by the limited sample size. The choice of respiratory specimen retrieval (BAL or non-directed-BAL) was led by the treating team and based on patient stability. Unfortunately, these data were not recorded for all study sites. While we recognise concerns regarding differing pathogen yield exist and could introduce bias, an increasing number of studies have shown non-directed BAL to be an effective surrogate [45, 46]. Another important consideration is the lack of quantitative PCR (qPCR). This would have helped determine the interference of pathogen absolute abundance and effect of contaminates in samples with a lower biomass. Saline negative controls were included to better understand the impact of background contamination; however, while these samples showed minimal contamination, we recognise that the low number of controls and read count achieved makes determination of the source difficult. As such, a degree of uncertainty remains regarding the origin of contamination and caution is advised in the interpretation of the negative controls. An approach that utilises both qPCR and the inclusion of controls from a variety of sources is better suited for future 16s sequencing studies [30, 47].

Data regarding antibiotic initiation were not available for this analysis; consequently, patients may have received antibiotics before BALF was collected. However, given that BALF recovery was to be done within the first 24 h, even if the patients received a dose of antibiotics just prior to sampling, it is less likely to impact bacterial yield [48]. However, we were unable to confirm this and the bacterial yield from conventional culturing might be negatively biased. The inclusion of such patients was a pragmatic approach and more reflective of clinical practice. Nonetheless, caution should be taken in relation to the interpretation of this data. Consideration should also be given to our reference standard (BALF culture ≥ 10^4^ CFU/ml) to dichotomise patients. We recognise that the use of microbiological diagnosis in isolation is imperfect and that true VAP diagnosis in practice often relies upon more, namely clinical suspicion in combination with a thorough clinical examination and simultaneous microbiological culture. However, capturing and standardising clinical decisions such as these is inherently difficulty, particularly across multiple study sites. Consequently, quantitative culture while not perfect remains the most commonly used reference standard to evaluate novel index tests in VAP research [49]. Additionally, the inclusion of only patients with a clinical suspicion strengthens our standard and better reflects clinical practice; however, we recognise that a negative BALF culture might mis-represent patients and not definitively exclude VAP, especially if they have received antibiotics.

The results of this study imply that while bacterial composition and diversity derived from 16s rRNA analysis differ between patients suspected of VAP with and without positive BALF culture, its place in clinical practice is not guaranteed. Targeted multiplex PCRs that offer rapid and early pathogen detection [50–52] and real-time metagenomic sequencing of respiratory specimens [53] challenge it and may further advance the role of culture-independent assessment of pathogen presence in the lungs. These platforms have shown promising pathogen identification with increasing accuracy and reduced processing times that could be performed at the bedside. More recently, the advent of rapid full-length 16s rRNA gene nanopore sequencing using the MinION sequencer (Nanopore Technologies, Oxford) has shown that genus identification using 16s rRNA is possible within as little as 2 h [54]. However, while encouraging, caution is needed if 16s sequencing is to be used as a truly agnostic tool. Instead, an approach reliant on pathogen-specific relative abundance thresholds could provide a better platform for clinical integration if used in a “rule out” fashion. Further prospective studies and replication in larger cohorts are needed first. However, the continued decreasing cost of metagenomics, reduced taxonomic resolution of 16s rRNA sequencing and lack of antibiotic susceptibility are likely to limit this role, with truly agnostic and unbiased metagenomic sequencing at the beside superseding it.

## Conclusion

In conclusion, patients who were suspected of VAP with a positive culture had increased dysbiosis and genus dominance compared to patients with negative cultures. An increased inflammasome caspase-1-dependent IL-1b release was associated with a reduced species evenness and increased pathogenic bacterial presence, providing a possible causal link between microbiome dysbiosis and lung injury development. However, measures of diversity were an unreliable predictor of culture positivity and 16s sequencing used in an unbiased capacity showed limitations for pathogen identification, that may be overcome if pathogen-specific relative abundance thresholds are used.

## Supplementary Information

**Additional file 1**.** Table S1**. Patient characteristics of all patients suspected of VAP during study period. Table S2. Absolute counts shown for the top genera identified from control samples.** Figure S1**. Study flow chart. Figure S2. Comparative absolute count analysis showing genera identified in BALF samples compared to negative control samples. Figure S3. The top 10 identified genera enriched in BALF negative specimens, shown for both negative and positive BALF cultures. Microbiota analysis. Library Preparation & 16s rRNA sequencing analytical pipeline.

## Data Availability

The data sets generated and/or analysed during the current study are available at the NCBI (SRA accession no. PRJNA780512) repository [https://www.ncbi.nlm.nih.gov/bioproject/PRJNA780512/]. Reproducible code for data analyses and figures is available and can be provided on request.
