# Supplementary material for: Composition and diversity analysis of the lung microbiome in patients with suspected ventilator-associated pneumonia
Source: Crit Care. 2022 Jul 6;26:203. doi: 10.1186/s13054-022-04068-z (PMC9261066; doi:10.1186/s13054-022-04068-z)
Supplement: Supplementary file 1 — Additional file 1. Table S1. Patient characteristics of all patients suspected of VAP during study period. Table S2. Absolute counts shown for the top genera identified from control samples. Figure S1. Study flow chart. Figure S2. Comparative absolute count analysis showing genera identified in BALF samples compared to negative control samples. Figure S3. The top 10 identified genera enriched in BALF negative specimens, shown for both negative and positive BALF cultures. Microbiota analysis. Library Preparation & 16s rRNA sequencing analytical pipeline. [file 13054_2022_4068_MOESM1_ESM.docx]

**Additional file 1**

**Composition and diversity analysis of the lung microbiome in patients with suspected ventilator-associated pneumonia**

Authors:

Dominic Fenn [1], Mahmoud I. Abdel-Aziz [1], Pouline M. P. van Oort [2], Paul Brinkman [1], Waqar M. Ahmed [4], Timothy Felton [4], Antonio Artigas [4], Pedro Póvoa [5], Ignacio Martin-Loeches [6], Marcus J. Schultz [7], Paul Dark [4], Stephen J. Fowler [4] and Lieuwe D.J. Bos [7] on behalf of the BreathDx Consortium

**Content:**

- Tables: S1-S2
- Figures: S1-S3
- Methods: Microbiota analysis

**Table S1:** Patient characteristics of all patients suspected of VAP during study period (N=108).

|  | **Included (N=90)** | | **Excluded (N=18)** | |
| --- | --- | --- | --- | --- |
|  | **Culture negative**  **(N = 53)** | **Culture positive**  **(N = 37)** | **Culture negative**  **(N=10)** | **Culture positive**  **(N=8)** |
| **Age, median (IQR) yrs** | 59.0 (48-68) | 56.5 (38.0-66.5) | 59.5 (49.3-65.3) | 63 (59.8-67.8) |
| **Male, n (%)** | 35 (66.0) | 23 (62.2) | 8 (80) | 6 (75) |
| **Days on MV^*^, median (IQR)** | 8 (4.5-12.5) | 7.0 (5.0-10.0) | 9.5 (6.3-15) | 6.0 (4.0-9.3) |
| **Admission type, n (%)** |  |  |  |  |
| Medical | 29 (54.7) | 13 (35.1) | 6 (60) | 2 (25) |
| Planned surgical | 10 (18.9) | 11 (29.7) | 1 (10) | 4 (50) |
| Emergency surgical | 13 (24.5) | 13 (35.1) | 3 (30) | 2 (25) |
| Unrecorded | 1 (1.9) | 0 | 0 | 0 |
| **Trauma, n (%)** | 12 (22.6) | 18 (48.6) | 2 (20) | 1 (12.5) |
| **Neurosurgery, n (%)** | 11 (20.8) | 12 (32.4) | 2 (20) | 2 (25) |
| **COPD, n (%)** | 8 (15.1) | 5 (13.5) | 0 | 1 (12.5) |
| **ARDS, n (%)** | 3 (5.7) | 0 (0.0) | 1 (10) | 0 |
| **APACHEII score, median (IQR)** | 21 (15-24) | 14 (10-20) | 18 (15-20.8) | 20 (14.8-22.5) |
| **CPIS score, median (IQR)** | 5 (4-6) | 7 (6-7) | 5 (4-5.8) | 7 (3.8-7.3) |
| **T_max_, ^o^C, median (IQR)** | 38 (37-39) | 38 (37-38) | 39 (37-39.8) | 38 (36-38) |
| **WCC, median (IQR) 10^9^/ml** | 15.5 (11.3-21.0) | 13 (11-17) | 14 (10-20) | 13 (11.5-13.5) |
| **PaO_2_/FiO_2_ _max_, median (IQR) mmHg** | 240 (180-283.5) | 232.5  (168.8-283.1) | 160  (119.0-260.0) | 233.5  (197.8-270) |
| **P_max_, median (IQR) cmH_2_O** | 20 (16-25) | 21 (16-24) | 19 (16-25) | 22 (18.3-30.5) |
| **PEEP, median (IQR) cmH_2_O** | 8 (5-10) | 8 (5-10) | 5 (5-10) | 5(5.0-5.5) |
| **Tidal volume, median (IQR) ml** | 479 (429.5-580) | 538  (449-620) | 415  (360-473) | 442.5  (374.3-556.5) |
| **Genus culture results****, n **(%)** |  |  |  |  |
| Enterobacter |  | 2 (5.4) |  |  |
| Escherichia |  | 2 (5.4) |  | 1 (12.5) |
| Haemophilus |  | 4 (10.8) |  | 1 (12.5) |
| Klebsiella |  | 4 (10.8) |  | 2 (25) |
| Pseudomonas |  | 10 (27.0) |  |  |
| Serratia |  | 1 (2.7) |  | 1 (12.5) |
| Staphylococcus |  | 13 (35.1) |  | 2 (25) |
| Stenotrophomonas |  | 1 (2.7) |  | 1 (12.5) |
| **ICU LOS, median (IQR) days** | 18 (14.0-26.8) | 22 (16- 35) | 33 (26.0-36.8) | 17 (9.0-21.3) |
| **Hospital LOS, median (IQR) days** | 26.5 (15.5-42.8) | 34 (21.8-64.5) | 33 (24-52) | 19 (14.0-39.5) |
| **ICU mortality, n (%)** | 12 (22.6) | 4 (10.8) | 6 (60) | 4 (50) |

﻿**Days on mechanical ventilation (MV) until VAP suspicion. ^**^Potentially >1 cultured pathogen per patient. ARDS, acute respiratory distress syndrome; APACHE, Acute Physiology and Chronic Health Evaluation; CPIS, Clinical Pulmonary Infection Score; Tmax, maximum temperature; WCC, white cell count; FiO2 max, maximum inspired fraction of oxygen ratio; Pmax, maximum airway pressure; PEEP, positive end- expiratory pressure; LOS, length of stay*

**Table S2:** Absolute counts shown for the top genera identified from control samples (saline wash).

|  |  | **Control 1** | **Control 2** |
| --- | --- | --- | --- |
|  | **Total sample count** | **24** | **39** |
| **Phylum** | **Genus** |  |  |
| Proteobacteria | Neisseria |  | 22.0 |
| Proteobacteria | Aquabacterium | 12.0 |  |
| Proteobacteria | Klebsiella | 10.0 |  |
| Actinobacteria | Actinomyces |  | 5.0 |
| Proteobacteria | Cronobacter |  | 5.0 |
| Firmicutes | Streptococcus |  | 4.0 |
| Proteobacteria | Massilia |  | 3.0 |
| Other | EU488070_g | 2.0 |  |

**Figure S1:** Study flow chart


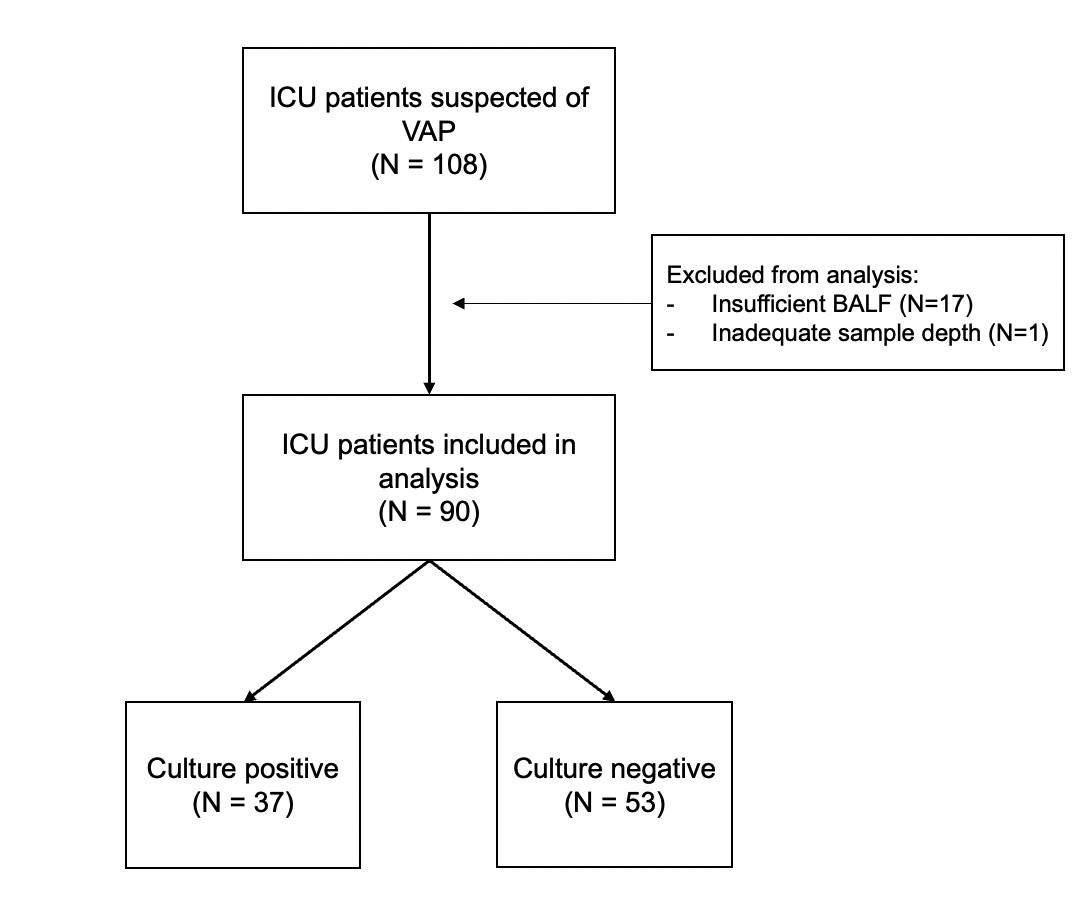


**Figure S2:** Comparative absolute count analysis showing genera identified in BALF samples compared to negative control samples (saline wash) along with total bacterial count for all BALF samples (n=90, mean = 10599) and the control samples (Control 1 = 24, Control 2 = 39). Control samples show little to no detectable reads suggesting minimal bacterial contamination.

**Figure S3:**

The top 10 identified genera enriched in BALF negative specimens. The bacterial genera are ranked in descending order of mean relative abundance with errors bars representing standard deviation and shown for both negative and positive BALF cultures

**Microbiota analysis**

DNA extraction from BALF was done using the PowerFecal DNA Kit (Qiagen, Venlo, Netherlands). Sequencing was performed using the Illumina MiSeq platform (CGEB-Integrated Microbiome Resource, Halifax, Canada) in line with manufacturer’s instructions. The DADA2 pipeline was used for further processing.

**Library Preparation**

*Adapted from Integrated Microbiome Resource (IMR) – Protocols*

*Available from: http://imr.bio/protocols.html*

Amplicon fragments were PCR-amplified in duplicate using separate template dilutions (generally 1:1 & 1:10) using the high-fidelity Phusion polymerase. A single round of PCR was done using primer pair 515F/ 926R to target various sub-regions of the 16s V4-V5 gene with multiplexing which allows up to 380 samples per run. PCR products were then verified visually using automated agarose gel electrophoresis (Nimbus Select Hamitlon, Reno, USA and Coastal Genomics, Burnaby, Canada). Any identified samples with failed PCRs were re-amplified. Ahead of sequencing, the PCR reactions from the same samples were pooled in one plate, then cleaned-up and normalized using the high-throughput Just-a-Plate 96-well Normalization Kit (Charm Biotech, California, USA) before being quantified fluorometrically. Paired-end reads from the targeted amplicon sequencing were quality checked using FastQc and MultiQC (1) before primers and adapters sequences were removed for amplicon purification using Cutadapt (2).

**16s rRNA sequencing analytical pipeline**

﻿Sequencing was performed using CGEB-Integrated Microbiome Resource pipeline that works to construct amplicon sequence variants (ASVs) before assigning taxonomic classification through the use of publicly available databases. This is done through several steps including: (1) quality filtering and trimming; (2) dereplicating sequences; (3) learning dataset-specific error rates; (4) denoising and removing potential errors sequences; (5) merging paired-end reads and removing mismatches; and finally (6) removing chimera. Internal technical controls, including both negative controls (blanks) and positive controls (mock communities) were part of the quality control sequencing process (3). In addition to this, negative control samples were included containing saline solution that was stored, processed and sequenced as a patient sample. These showed little to no detectable reads implying that there was minimal bacterial contamination (Figure S2 and table S2). The publicly available database EzBioCloud (version May 2018) (4) was selected to assess taxonomic classification of the identified ASVs since it is an integrated database, allowing for improved taxonomic identification.

**References**

1. Pfeifer SP. From next-generation resequencing reads to a high-quality variant data set. Heredity (Edinb). 2017;118(2):111–24.

2. Martin M. Cutadapt removes adapter sequences from high-throughput sequencing reads. EMBnet.journal. 2011 May;17(1):10.

3. Comeau AM, Douglas GM, Langille MGI. Microbiome Helper: a Custom and Streamlined Workflow for Microbiome Research. mSystems. 2017;2(1).

4. Yoon SH, Ha SM, Kwon S, Lim J, Kim Y, Seo H, et al. Introducing EzBioCloud: A taxonomically united database of 16S rRNA gene sequences and whole-genome assemblies. Int J Syst Evol Microbiol. 2017;67(5):1613–7.
